# Antioxidant Phytochemicals at the Pharma-Nutrition Interface

**DOI:** 10.1155/2017/6986143

**Published:** 2017-11-08

**Authors:** Elena Azzini, Jasminka Giacometti, Gian Luigi Russo

**Affiliations:** ^1^Council for Agricultural Research and Economics (CREA), Research Center for Food and Nutrition, Via Ardeatina 546, 00178 Rome, Italy; ^2^Department of Biotechnology, University of Rijeka, Radmile Matejčić 2, 51000 Rijeka, Croatia; ^3^Institute of Food Sciences, National Research Council, 83100 Avellino, Italy

Noncommunicable diseases (NCDs), including obesity, diabetes, cardiovascular diseases, and cancer, represent an emerging global health issue. Nutrition, as known, represents one of the most important aspects of health; several researchers have shown that nutrition plays a crucial role in the prevention of food deficiencies, behavioural disorders, and chronic diseases. The last decades saw the proliferation of studies on the healthy benefits of specific classes of nutrients. Among these, phytochemicals took the lead for their capacity to act as antioxidants, a function which inspired the so-called “antioxidant hypothesis” or “free radical theory” for degenerative diseases originated back in the late 1980s–middle 1990s. After more than twenty years of intense researches, phytochemicals remain an *éminence grise* in the area of the natural remedies against degenerative diseases. In fact, if, from one side, their functional pleiotropy guarantees multiple therapeutic and preventive effects, their low bioavailability and high metabolic transformation represent an unsolved issue on the way to demonstrate a clear structure-function relationship in regulating cellular physiology.

The aim of the present special issue was not to propose a solution for all the questions surrounding the mechanisms of action of phytochemicals but to bring the attention of the readers on specific themes with a selection of high quality research articles and reviews, hoping to stimulate their thinking. This special issue comprising nine papers is focused on various aspects of phytochemicals effects when administered as extracts in animal and cellular models. The articles have been selected on the basis of fundamental ideas/concepts rather than the thoroughness of techniques employed. The papers are organised as follows.

D. de Almeida Bauer Guimarães et al. suggest that pitaya extract (PE) may have a protective effect against breast cancer. PE showed high antioxidant activity, and high values of anthocyanins induced a selective decrease in cell proliferation caused by PE in MCF-7 (ER^+^) cell line and an increase in G_0_/G_1_ phase followed by a decrease in G_2_/M phase. Also, PE induced apoptosis in MCF-7 (ER^+^) cell line and suppressed BRCA_1_, BRCA_2_, PRAB, and Er*α* gene expression.

D. Załuski et al. highlighted the fruits of *Eleutherococcus* species rich in polyphenols as a new income source of agriculture and industry in natural products and foods. These fruits act as antioxidants, induce apoptosis in Jurkat 45 leukemic cell line, and inhibit the activity of MMP-1, MMP-2, MMP-3, and MMP-9.

L. Song et al. reported a preclinical systematic review about G-Rg1 as a promising potential neuroprotective against PD model through different mechanisms including antineuroinflammatory, antioxidative, and antiapoptotic effects. On the other hand, a letter by Yi-bo et al. expressed comment on “A Preclinical Systematic Review of Ginsenoside-Rg1 in Experimental Parkinson's Disease” by inviting the authors to a more conservative conclusion. Thus, further controlled studies in animals should be attempted to establish the G-Rg1 as a drug candidate, rather than confirmed by clinical trials immediately.

V. R. Pasupuleti et al. have given a review of several health benefits of honeybee products used as food, such as honey, propolis, and royal jelly, on metabolic diseases, cancers, and others diseases.

T. Bonamigo et al. described how the chemical constituents of propolis, such as phytosterols, terpenes, aromatic acids, and tocopherol, are important in the control of diseases. In the study of antioxidant, cytotoxic, and toxic activities of ethanol extracts of propolis (EEPs) obtained from the Brazilian stingless bees *Scaptotrigona depilis* (EEP-S) and *Melipona quadrifasciata anthidioides* (EEP-M), they indicated the cytotoxic activity of EEPs against erythroleukemic cells and necrosis as the main mechanism of death observed. In addition, cytotoxic doses of EEPs were not toxic against *Caenorhabditis elegans*. Bioactive mixture found in EEP-S and EEP-M can be used in the control of diseases associated with oxidative stress and tumour cell proliferation.

Z. Tuzcu et al. showed that cinnamon reduces the hyperlipidemia, inflammation, and oxidative stress through activating transcription factors and antioxidative defense signaling pathway in high-fat diet- (HFD-) fed rats. Cinnamon polyphenol also suppressed the expression of hepatic sterol regulatory element-binding protein-1c (SREBP-1c), liver X receptor (LXR-*α*), ATP-citrate lyase, fatty acid synthase (FAS), and nuclear factor kappa B p65 (NF-*κ*B p65) and enhanced the peroxisome proliferator-activated receptor alpha (PPAR-*α*), insulin receptor substrate 1 (IRS-1), nuclear factor-E2-related factor-2 (Nrf2), and heme oxygenase 1 (HO-1) expressions in the HFD rat livers.

J.-T. Liu et al. investigated the molecular mechanism supporting the protective effect of red yeast rice (RYR; *Monascus purpureus*-fermented rice) in limiting the vascular complications of diabetes. Using an ex vivo cellular model represented by human bone marrow-derived proangiogenic cells (PACs) isolated from healthy donors and treated with nontoxic concentrations of RYR extract (<50 *μ*g/ml), the authors demonstrated its capacity to induce time-dependently Nrf2 nuclear translocation in PAC cells which resulted in a dose-dependent induction of HO-1 mRNA expression and, in parallel, a dose- and time-dependent increase in HO-1 protein expression. Using multiple and complementary approaches, the authors confirmed the beneficial effects of RYR extract showing its ability to inhibit senescence and oxidative stress (production of ROS) following treatment of PAC cells with high glucose (30 mM). Although the direct target of RYR, which triggers the cascade of reactions, leading to the activation of HO-1, has not been identified, Chen's group optimistically proposes that RYR extract may serve as alternative and complementary medicine in decreasing the vascular complications of diabetes.

A. Molfino et al. analyze the active, anti-inflammatory role of the specialized proresolving mediators (SPMs), defined as a family of lipid mediators derived from omega-6 and omega-3 polyunsaturated fatty acids (PUFA) and including well-known bioactive compounds, such as lipoxins (omega-6 PUFA derived) and D-/E-series resolvins, protectins, and maresins (omega-3 PUFA derived). The authors focused on the anti-inflammatory and proresolving role of omega-3-derived SPM in critical illnesses. They firstly reviewed the importance of genetic signature in determining the level of expression of SPMs, their biosynthetic isomers, and pathways in trauma patients. Although still circumstantial, literature data suggest that circulating levels of PUFAs and PUFA metabolites may influence the inflammatory responses via the NF-*κ*B pathway. It remains to be determined the cause-effect relationship between severe inflammatory pathological conditions and inactivation of specific SPM pathways. The anti-inflammatory or proresolving effects of PUFAs in clinics are encouraging since the latest clinical meta-analysis suggests a reduced ICU length of stay in patients following cardiac surgery and supplemented with omega-3 PUFA-enriched parenteral emulsions. A. Molfino et al. conclude that evidence are increasing in favor of a protective role of omega-3 PUFA-derived SPMs as anti-inflammatory and proresolving nutrients, although future work must be devoted to assess the optimal lipid emulsions and their specific uses.

E. Azzini et al. highlighted the following key points: the healthy relationship between anthocyanin supplementation and their antiobesity effects suffers of the same contradictions and doubtable interpretations, which emerge when the beneficial responses of other phytochemicals towards different degenerative diseases are considered; the different dosage applied in animal versus clinical studies; the complex metabolism and biotransformation to which anthocyanins and phytochemicals are subjected in the intestine and tissues; the possibility that different components present in the supplemented mixtures can interact generating antagonistic, synergistic, or additive effects difficult to predict; and the difference between prevention and therapy. The authors concluded that the evolution of the field must seriously consider the need to establish new and adequate cellular and animal models, which may, in turn, allow the design of more efficient and prevention-targeted clinical studies.

Long duration and slow progression of NCDs could be counteracted by modulating the intake of macro- and micronutrients. The use of food components (including polyphenols, glucosinolates, PUFAs, fibers, and friendly bacteria) among patients on conventional pharmacological therapy should be carefully assessed due to the possibility of food-drug interactions. Even if several food compounds may exert a prophylactic function within the human body, their bioavailability and bioactivity have high interindividual variability and the mechanisms of biological action of food extracts and bioactive compounds remain to be elucidated. Consequently, well-designed randomized clinical trials are needed to clarify the precise role of food components and/or new food in human health.

